# Meningeal Hemangiopericytoma Presenting as Pure Gerstmann Syndrome: A Double Rarity

**DOI:** 10.7759/cureus.15863

**Published:** 2021-06-23

**Authors:** Prashant Natteru, Lakshmi Ramachandran Nair, Gustavo Luzardo, Nawal Shaikh

**Affiliations:** 1 Neurology, University of Mississippi Medical Center, Jackson, USA; 2 Pathology, University of Mississippi Medical Center, Jackson, USA; 3 Neurological Surgery, University of Mississippi Medical Center, Jackson, USA; 4 Neuro-Oncology, University of Mississippi Medical Center, Jackson, USA

**Keywords:** gerstmann syndrome, hemangiopericytoma, solitary fibrous tumor, dural mass, neuro oncology

## Abstract

Gerstmann syndrome is a neurobehavioral syndrome characterized by four cardinal symptoms: acalculia, agraphia, finger-toe agnosia, and dysgraphia. The syndrome is caused primarily by lesions at the confluence of parietal, temporal, and occipital lobes, but also can involve the middle frontal lobe of the dominant hemisphere. Documented inciting lesions include stroke, tumor, hemorrhage, arteriovenous malformations, and seizures. A meningeal solitary fibrous tumor (SFT)/hemangiopericytoma (HPC) is a diagnostic challenge due to its resemblance to more common brain tumors like meningioma, with histopathology being the definitive diagnostic test. A 37-year-old male presented to our tertiary center with blurred vision, “not being himself,” and “acting funny” for three weeks. On exam, he was found to have a right inferior quadrantanopia, grade II papilledema and demonstrated all four symptoms of Gerstmann syndrome - inability to perform simple calculations (acalculia), or identify his fingers (finger agnosia), could not distinguish his left side from the right (left-right disorientation), nor write out his name (agraphia). Brain imaging showed an extra-axial, highly vascularized 7.6-cm mass compressing the left parietal lobe. He underwent a complete resection of the mass. Postoperatively, he had gradual improvement with complete resolution of agraphia, acalculia, finger agnosia, and left-right disorientation within a week status post-resection. Tumor pathology indicated hemangiopericytoma/solitary fibrous tumor. This case enunciates the enigmatic tetrad of Gerstmann syndrome. Though classically described as a sequela of stroke, the mass effect of the tumor on the parietal lobe may produce the symptoms, which can resolve following resection.

## Introduction

Meningeal solitary fibrous tumors (SFT)/hemangiopericytomas (HPC) are rare and aggressive mesenchymal neoplasms arising from the pericytes of Zimmerman, which represent <2.5% of all meningeal tumors and 1% of primary central nervous system tumors [1]. Pure Gerstmann syndrome is a neurobehavioral syndrome characterized by four cardinal manifestations: acalculia (loss of the ability to perform arithmetical operations and use numerical concepts), agraphia (acquired disturbance in the ability to write), finger agnosia (inability to distinguish, name, and recognize the fingers), and right-left disorientation (right-left discrimination defect when using language) [2]. Both these entities are exceedingly rare. We describe a case of meningeal HPC presenting as pure Gerstmann syndrome.

## Case presentation

A 37-year-old right-handed male with no known past medical history presented to the emergency department with headache, blurred vision, “not being himself,” and “acting funny” for about three weeks before presentation. The family described his behavior as "not being himself and acting funny" as he was unable to write out his name or perform simple mathematics. He was seen by an optometrist for blurred vision who noticed bilateral papilledema and referred him to an outside facility for workup. At the outside facility, he underwent brain imaging and was transferred to our center for further workup. His neurological examination was remarkable for a holocephalic headache, right inferior quadrantanopia, grade II papilledema, inability to perform simple calculations (acalculia), or name/identify his fingers (finger agnosia) could not distinguish his left side from the right (right-left disorientation), nor write out his name (agraphia).

**Figure 1:**
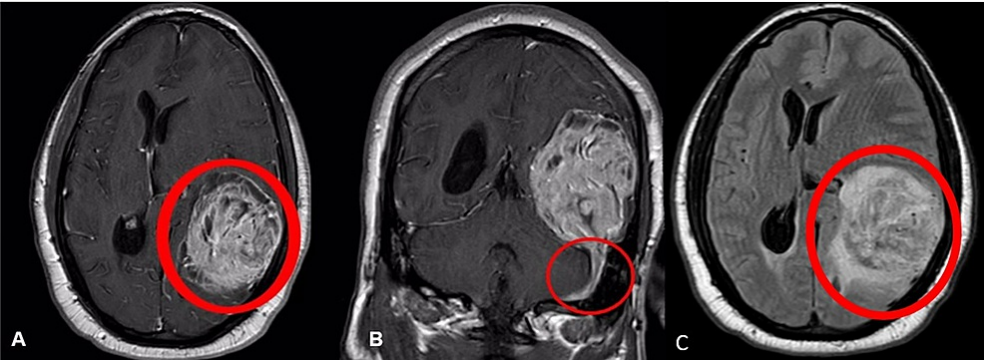
Magnetic resonance imaging (MRI) of the brain (T1 axial sequence with contrast) showing (A) an extra-axial, heterogeneous, highly vascularized 7.6-cm mass compressing the left parietal lobe along with a centimeter left-to-right midline shift (encircled). (B) MRI brain T1 coronal sequence with contrast demonstrating the dural tail (encircled). (C) MRI T2-fluid attenuated inversion recovery (FLAIR) sequence showing an alternating pattern of hypo-intensity and hyperintensity ("yin-yang" sign) along with edema.

Results of basic laboratory testing were unremarkable, including a complete blood count and electrolytes. Computed tomography (CT) scan of the head from the outside facility showed a heterogeneous intracranial mass in the left temporoparietal region with a left-to-right 1-cm midline shift. Magnetic resonance imaging of the brain with and without gadolinium contrast showed an extra-axial, neovascularized, avidly enhancing 7.6-cm mass with multiple foci of cystic transformation, and a dural tail (Figures 1A, 1B).

This mass was compressing the left temporoparietal lobes resulting in a left-to-right midline shift of 1 cm and effacement of the left lateral ventricle. An electroencephalogram showed mild theta slowing in the left cerebral hemisphere. Based on the imaging, neurosurgery was consulted for diagnostic/therapeutic intervention. A digital subtraction angiogram of the head and neck showed the left middle meningeal artery (MMA) as the feeder artery to the mass.

**Figure 2:**
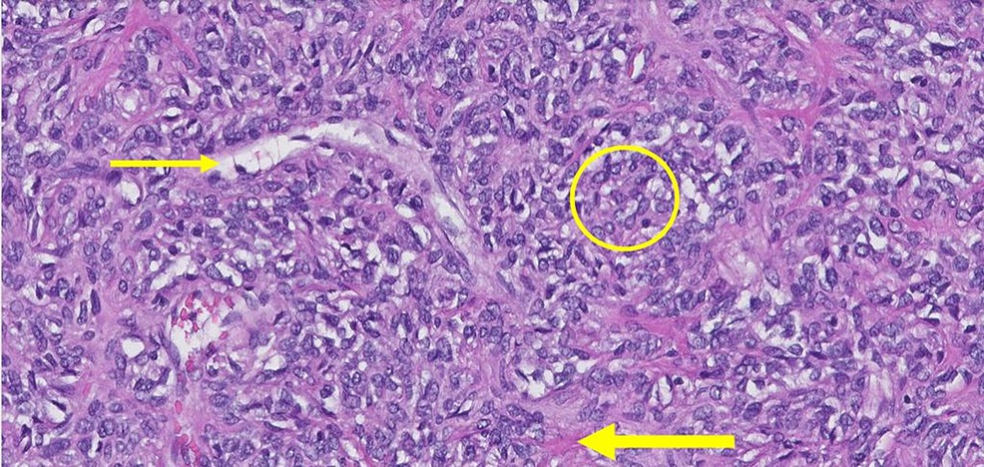
Photomicrograph (H&E stain 100×) showing a cluster of oval fibroblastic cells (encircled) intermixed with pink collagenous stroma (thick arrow) and stag-horn-shaped blood vessel (thin arrow). H&E - Hematoxylin and eosin

Before the planned surgical resection, he underwent a transcatheter arterial embolization to the left MMA, after which a left-sided craniotomy was performed along with near-total surgical resection of the dural-based mass. This was then sent for histopathological diagnosis, which revealed oval fibroblastic cells intermixed with pink collagenous stroma and “stag-horn” shaped blood vessels suggestive of a solitary fibrous tumor (WHO Grade II) / hemangiopericytoma (Figure 2).

Postoperatively, he had gradual improvement with complete resolution of agraphia, acalculia, finger agnosia, left-right disorientation, headache, right inferior quadrantanopia, and papilledema within a week of resection. He was seen in the Neuro-oncology clinic about eight weeks from discharge with a normal neurological examination, and a repeat brain MRI showed no recurrence of the tumor. Also, CT neck, chest, abdomen, and pelvis along with skeletal scintigraphy did not reveal any metastases.

## Discussion

Gerstmann syndrome is caused primarily by lesions at the confluence of parietal (angular gyrus), temporal and occipital lobes, but also can involve the middle frontal lobe of the dominant hemisphere [3]. The underlying pathophysiology can be attributed to a dual theoretical explanation: (a) it is a disconnection syndrome between the cortical and subcortical regions and (b) the angular gyrus just processes the information underlying the Gerstmann tetrad, and damage to it results in abnormal verbally mediated spatial knowledge [3]. Pure Gerstmann syndrome (complete tetrad) is a rarity, as it usually appears as an “incomplete tetrad” of symptoms or in association with other cognitive deficits like apraxia, alexia, semantic aphasia, disorders of spatial orientation, and word-finding difficulties [4]. Etiology varies from more common strokes (ischemic, hemorrhagic), space-occupying lesions like tumors, to less common epilepsy.

Meningeal SFT and HPC were considered different entities until 2016, but after the discovery of chromosome 12q13 inversion causing NAB2-STAT6 fusion, 2016 World Health Organization classification defined these tumors as a single entity [5]. These tumors commonly affect men in the fourth-fifth decade, with the clinical features based on the tumor location [6]. SFT/HPC tend to have more local relapses, but the distant spread is not uncommon [5,6].

Radiologically, SFT/HPC on MRI is isointense on T1-weighted images and heterogeneously hypointense on T2-weighted images [4,6,7]. At times, there can be an alternating pattern of hypo-intensity and hyperintensity on postcontrast T1-weighted images, commonly referred to as the “yin-yang” sign. This sign reflects the staghorn vascularity and the cellular variability in the different areas of SFT/HPC [6]. These tumors can mimic other meningeal tumors like meningioma on imaging, and thus the histopathological diagnosis holds the key.

Histological grading is based on phenotype and mitotic count. SFT-type phenotype with no increased mitosis is classified as Grade 1. Tumors with HPC phenotype are further classified as Grade 2 or 3 depending on the number of mitoses. Less than five mitoses per 10 high power fields are Grade 2 and more than five mitoses per 10 high power fields would be Grade 3 [8]. In the SFT variant, cells with a spindle to ovoid nucleus are arranged in a haphazard pattern or short fascicles with hyper and hypocellular areas and thick bands of collagen, while the HPC variant is usually more cellular with a round to ovoid nucleus, little intervening stroma with reticulin network [9]. The presence of characteristic, thin-walled branched blood vessels, described as staghorn blood vessels are seen in both phenotypes. Immunohistochemical stains (characterized by positive expression of mainly STAT6 but also CD34 and vimentin) can aid in confirming the diagnosis [6]. Our patient’s specimen showed a cellular tumor with HPC phenotype with a round to oval nucleated cells. The intervening stroma was collagenous but scant. Multiple characteristic blood vessels were present. Areas with up to three mitoses per 10 high power fields were identified making it a Grade 2 tumor.

A multimodal approach encompassing surgical resection followed by postoperative adjuvant radiotherapy for localized disease, but options are limited in metastatic disease. Of late, newer options like Pazopanib have been explored as targeted therapies [5,10].

## Conclusions

Our patient presented with clinical features of pure Gerstmann syndrome secondary to the mass effect of an underlying meningeal hemangiopericytoma, both of which are rare. This case enunciates the enigmatic tetrad of Gerstmann syndrome. Though classically described as a sequela of stroke, the mass effect of tumor-like HPC on the parietal lobe may produce the symptoms which can resolve following resection.
